# Musculoskeletal Radiology Education: A National Survey by the Italian College of Musculoskeletal Radiology

**DOI:** 10.3390/diagnostics14010040

**Published:** 2023-12-25

**Authors:** Domenico Albano, Stefano Fusco, Marcello Zappia, Luca Maria Sconfienza, Andrea Giovagnoni, Alberto Aliprandi, Carmelo Messina

**Affiliations:** 1 IRCCS Istituto Ortopedico Galeazzi, 20161 Milan, Italy; io@lucasconfienza.it (L.M.S.); carmelomessina.md@gmail.com (C.M.); 2 Dipartimento di Scienze Biomediche, Chirurgiche e Odontoiatriche, Università degli Studi di Milano, 20122 Milan, Italy; 3 Dipartimento di Scienze Biomediche per la Salute, Università degli Studi di Milano, 20122 Milan, Italy; stefano.fusco@unimi.it; 4 Department of Medicine and Health Sciences, University of Molise, 86100 Campobasso, Italy; marcello.zappia@unimol.it; 5 Varelli Institute, 80126 Naples, Italy; 6 Department of Radiology, University Hospital “Azienda Ospedaliera Universitaria delle Marche”, 60121 Ancona, Italy; andrea.giovagnoni@ospedaliriuniti.marche.it; 7 Department of Clinical, Special and Dental Sciences, Università Politecnica delle Marche, 60121 Ancona, Italy; 8 Radiology Unit, Zucchi Clinical Institutes Spa, 20900 Monza, Italy; a_aliprandi@yahoo.it

**Keywords:** training, education, musculoskeletal radiology, ultrasound, MRI, interventional radiology

## Abstract

Background: Our aim was to understand how musculoskeletal training is structured in Italian residency programmes and the needs of young trainees. Methods: We sent out an online questionnaire (17 questions) to Italian Society of Radiology residents and board-certified radiologists aged up to 39 years. Results: A total of 1144 out of 4210 (27.2%) members participated in the survey; 64.7% were residents and 35.3% were board-certified radiologists. Just 26.6% of participants had dedicated rotations for musculoskeletal training during their residency, although this percentage substantially increased in replies from northern Italy. One-fourth of residents had a scheduled period of musculoskeletal ultrasound. Most participants (76.3%) had <20 h per year of musculoskeletal lessons. The majority considered their musculoskeletal education poor (57.7%) or average (21.9%). According to 84.8% of replies, no dedicated training period about interventional musculoskeletal procedures was scheduled. Further, just 12.8% of residents took active part in such interventions. Nearly all participants believed that the musculoskeletal programme during residency needs to be improved, particularly concerning practices in ultrasound (92.8%), MRI cases interpretation/reporting (78.9%), and practice in ultrasound-guided interventional procedures (64.3%). Conclusions: Despite some differences in the structure of musculoskeletal education provided by different regions, there is a shared demand for improvement in musculoskeletal training.

## 1. Introduction

In the era of precision medicine, we are witnessing a constant shift toward more subspecialised physicians and clinical activities, which is something occurring in all fields of radiology, particularly in musculoskeletal (MSK) imaging [1,2,3]. This is probably due to the ongoing introduction of novel technologies, the improvements in knowledge, and the increasing expectations of other specialists for more and more accurate diagnosis through imaging [4,5,6,7,8]. Hence, the improved performance of musculoskeletal radiologists goes hand in hand with the increased demands and needs of other physicians. This evolution concerns both diagnostic and interventional radiology, with the latter involving more precise and new percutaneous interventions that enable us to treat patients non-invasively, in some cases also replacing surgical procedures [9,10,11,12,13,14]. The importance of a constant update and training is proven by a previous study, which revealed that musculoskeletal scans account for more than 70% of all magnetic resonance examinations performed in Italy [15]. In this setting, musculoskeletal education and training during residency is essential to guarantee high-quality radiology practice and research [4,16,17].

In Italy, there is no standardised residency programme throughout the national territory, with almost 40 schools of radiology that build up the educational residency programmes. However, the Italian Ministry of Education, Universities and Research, has provided prerequisites for residency training, including musculoskeletal training, with a minimal number of reports/procedures that should be performed by the resident each year and during the entire residency [18]. Hence, it is expected to see wide variations between different schools and regions in the ways that they teach and learn musculoskeletal radiology, with programmes that are also based on the local needs of a regional healthcare system. Indeed, the Italian health service is organised with integrated public and private healthcare providers on a regional basis, with planning and organisation being delegated to each region under the supervision of national governments that provide recommendations and requirements. In this setting, the Italian College of Musculoskeletal Radiology, a subsection of the Italian Society of Radiology (SIRM), comprises more than 2000 members (out of 11,000 SIRM members) and delivers musculoskeletal education to Italian radiologists with conferences, webinars, and position papers, supporting the activities of the schools of residency [19].

Recently, an international survey published by the Young Working Group of the European Society of Musculoskeletal Radiology (ESSR) has highlighted the significant differences in the structure of musculoskeletal training in different countries, reporting the emerging interest from young residents and the urgency of education improvement [20]. To date, no previous studies have thoroughly investigated the models of musculoskeletal education in the different Italian regions. Thus, the aim of this national survey was to understand how musculoskeletal training is structured in residency programmes and the needs of young trainees, to provide insights regarding possible new teaching methods and interventions to spread knowledge about musculoskeletal radiology.

## 2. Materials and Methods

### 2.1. Study Design

We did not need Institutional Review Board approval to publish these data, since no patients were involved in the study. The questionnaire was developed by the participating panelists using a consensus process, where new questions were proposed and agreed in consensus by 7 panel members who are actively involved in activities promoted by the Italian College of Musculoskeletal radiology. The questionnaire was focused on training and self-perception of young board-certified radiologists and residents involved in musculoskeletal imaging and interventional procedures to understand whether and what kind of gaps exist in training programmes. The poll was approved by the SIRM Board Committee on 21 April 2023. The questionnaire was disseminated via email to all SIRM members under the age of 40 years (4210 members) on 27 April 2023. After one week, another email was sent out as a reminder and the survey was closed on 7 May 2023. In line with previous surveys, we used the free online tool “Google Forms” (Google LLC, Mountain View, CA, USA) to create the questionnaire and collect answers [17,20,21]. This poll consisted of 17 questions, of which fifteen required unique answers or multiple-choice selections, whilst two questions requested entering a free-text response.

### 2.2. Data Analysis

Answers were collected via Google Forms and were managed in aggregated form to ensure anonymity. The dataset was analysed by two radiologists with previous expertise in survey studies (D.A. and C.M.). Descriptive statistics were used; data and response rates were expressed as means and percentages. A sub-analysis was performed that compared replies from the three Italian macroregions (north, central, and south and islands). We further analysed data related to the top five Italian regions with the highest response rates.

## 3. Results

A total of 1144 replies from 4210 (27.2%) SIRM members under 40 years were included in our analysis, of which 64.7% were from residents (mean age of 29.5 ± 2.6 years) and 35.3% from board-certified radiologists (mean age of 34.8 ± 3.9 years). The full list of questions and answers is reported in Table 1. The total number of answers, stratified by region of residency, is summarised in Table 2. The top five represented Italian regions of residency were Lombardia (14.2%), Sicilia (14.0%), Lazio (9.8%), Toscana (9.8%), and Veneto (9.5%) (Table 2). The most heterogeneous responses from these five regions and from the three Italian macroregions (north, central, and south and islands) are reported in Table 3 and Table 4, respectively.

Among the board-certified radiologists, 27.2% of them stated they worked at university hospitals, 55.9% at public hospitals, and 16.8% at private clinics, while 88.1% of residents were training at university hospitals, 10.5% at public hospitals, and 1.4% in private clinics. Just 26.6% of participants had dedicated rotations for musculoskeletal training during their residency, with only 14.6% having reported a period longer than 6 months. In almost two-thirds of cases (63.7%), residents rotated in the different musculoskeletal imaging modalities instead of being assigned to a specific modality. In this regard, just one-fourth of residents had a scheduled period for musculoskeletal ultrasound. The vast majority of participants (76.3%) had <20 h per year of musculoskeletal lessons during their residency and 65.1% referred to senior consultants or junior specialists for their training. Most participants considered their musculoskeletal education poor (57.7%) or average (21.9%) and almost all of them stated they would like to learn musculoskeletal radiology with routine daily reporting under supervision (92.3%). According to 84.8% of replies, there was no dedicated training period about interventional musculoskeletal procedures. Further, just 12.8% of residents took active part in such interventions. Nearly all participants believed that the musculoskeletal programme during residency needs to be improved, particularly concerning practice in ultrasound (92.8%), MRI cases interpretation/reporting (78.9%), and practice in ultrasound-guided interventional procedures (64.3%). Lastly, 78.2% of participants felt the need to or had to carry out a musculoskeletal training period in other national or international referral centres.

**Figure 1:**
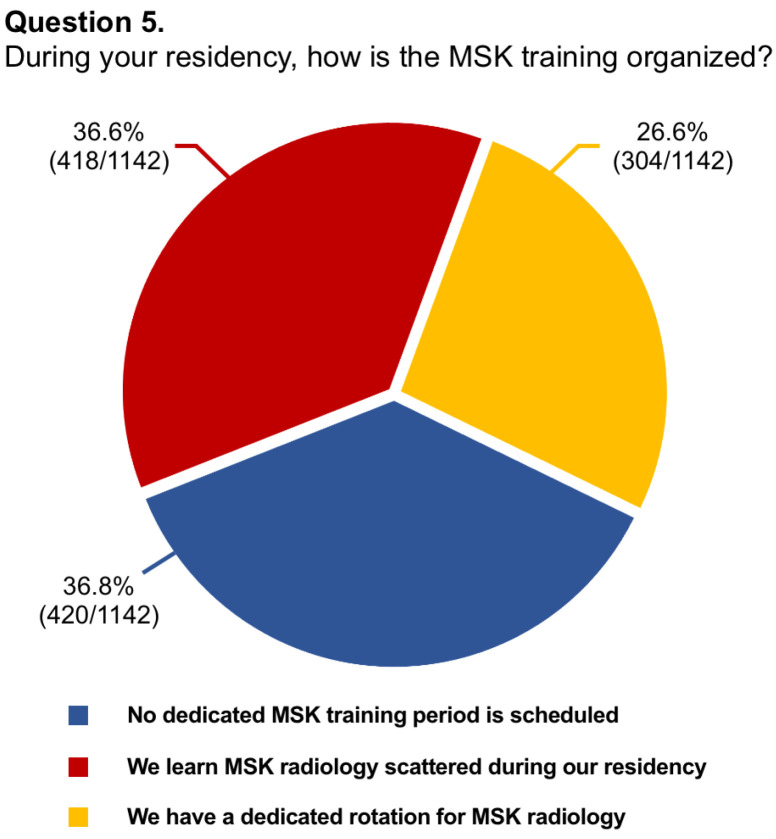
Graphical distribution of replies to question 5.

**Figure 2:**
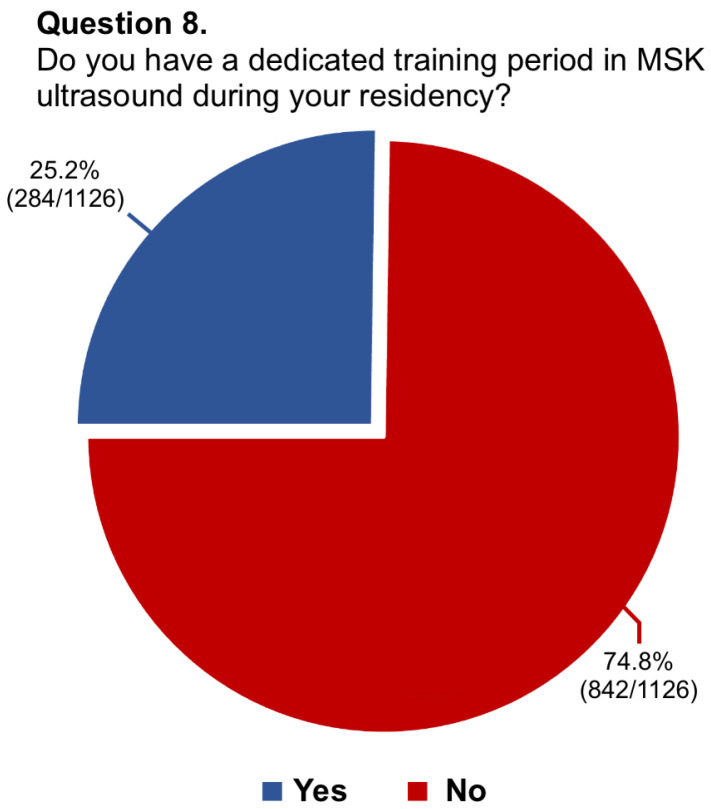
Graphical distribution of replies to question 8.

**Figure 3:**
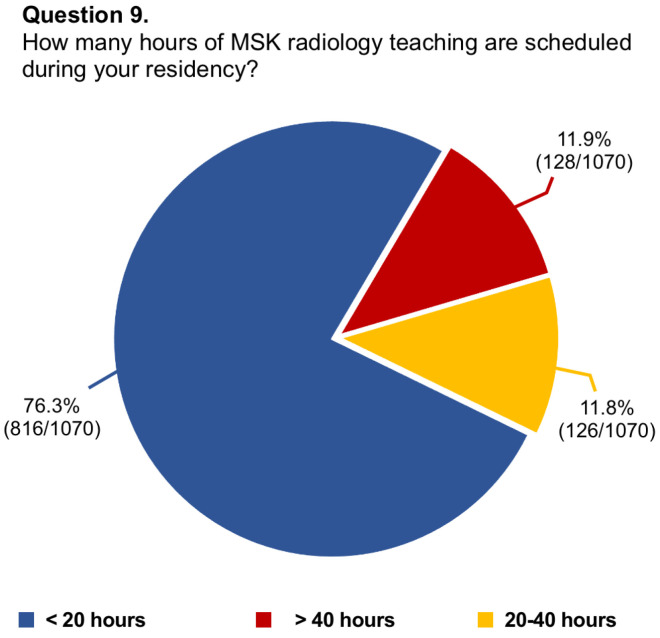
Graphical distribution of replies to question 9.

**Figure 4:**
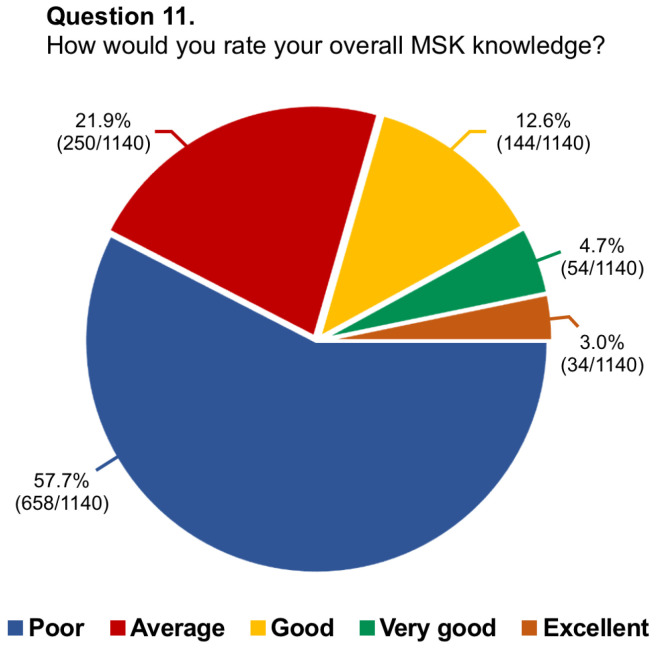
Graphical distribution of replies to question 11.

**Figure 5:**
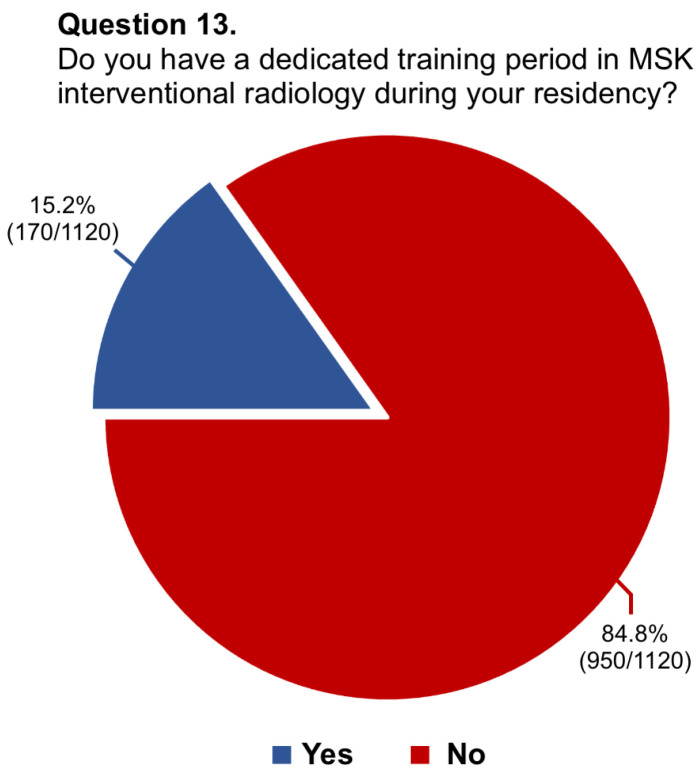
Graphical distribution of replies to question 13.

**Figure 6:**
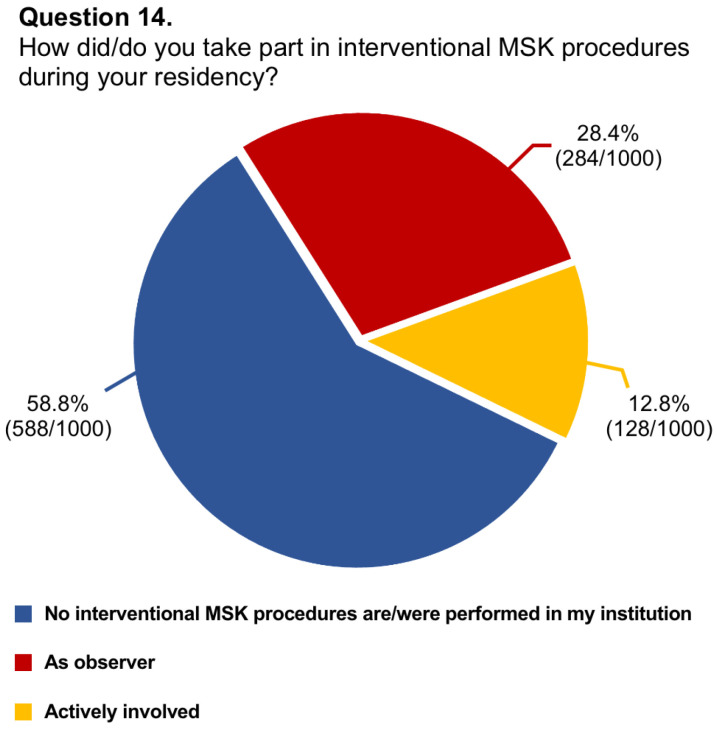
Graphical distribution of replies to question 14.

**Figure 7:**
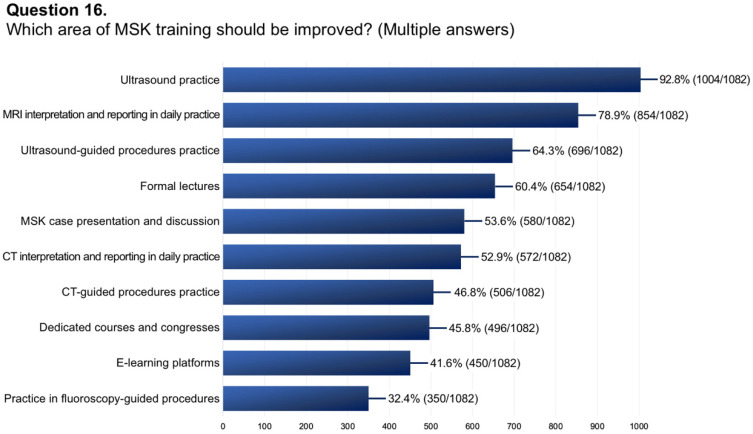
Graphical distribution of replies to question 16.

Questions #5 (structure of musculoskeletal training), #8 (dedicated training period of musculoskeletal ultrasound), #9 (allocation of hours to musculoskeletal radiology teaching), #11 (rating of musculoskeletal education), #13 (dedicated training period in musculoskeletal interventions), #14 (involvement in interventional procedures), and #16 (needs for improvement in musculoskeletal training) have been reported in graphics (Figure 1, Figure 2, Figure 3, Figure 4, Figure 5, Figure 6, and Figure 7, respectively).

## 4. Discussion

We have collected the replies of more than 25% of board-certified radiologists and radiology residents from different Italian regions who are SIRM members under the age of 40 years, thereby gathering an interesting report of education and training in musculoskeletal radiology across Italy. The main findings of this survey are the substantial heterogeneity of residency programmes in the different schools and the demands for improvement of radiology training, particularly for what concerns ultrasound and interventional procedures.

Almost two-thirds of responses were from residents; most of them were practicing in university hospitals and just a minority in public institutions, while a negligible number was working in private clinics. Regarding young board-certified radiologists, more than half of them were working in public hospitals and more of one-fourth in university hospital, which are preferred to private clinics, probably because the former are better geared to accommodate research, academia and access to more advanced technology.

Overall, our analysis shows that not enough space is guaranteed to musculoskeletal education in residency programmes. Indeed, dedicated musculoskeletal training has only been reported in about one-fourth of cases, mostly for a short time (less than six months). The lack of musculoskeletal education is also proven by the short period of musculoskeletal teaching included in residency programmes. A comprehensive and thorough musculoskeletal radiology education is essential for both general and sub-specialised radiologists, given that the former may deal with musculoskeletal examination in emergency departments or outpatient scans, while the latter must support clinical activity of highly specialised orthopaedists, rheumatologists, and other physicians in referral centres [22]. Notably, authors have highlighted that subspecialists are more accurate in about 80% of reports than general radiologists, particularly concerning oncologic examinations [3]; this is something that was also highlighted by Rozenberg and associates in reporting scans about musculoskeletal tumours [23]. Further, authors have shown that young trainees who underwent a dedicated musculoskeletal training period substantially improved their performance in bone densitometry interpretation, thereby impacting the start of osteoporosis treatment with drugs [24].

Residents mostly referred to senior or junior colleagues for their training, probably due to their preference to learn clinical practice and reporting under the supervision of more experienced consultants, rather than frontal lessons, e-learning platform, and courses. When dedicated musculoskeletal training periods were planned, residents generally were not assigned to a specific imaging modality. Further, the vast majority of them had not undergone a dedicated training period focused on musculoskeletal ultrasound and image-guided interventional procedures, in which young trainees have been actively involved in about 10% of cases. In fact, the majority of participants rated their musculoskeletal education poor-to-average and believed their residency programme should be improved, particularly concerning ultrasound, MRI cases interpretation/reporting, and practice in interventional procedures. The urge of improvement of musculoskeletal training programmes is also proven by the huge number of young board-certified radiologists and residents (almost 80%) that stated to need or had to carry out a training period in national or international referral centres for musculoskeletal imaging. Ultrasound plays a crucial role in musculoskeletal imaging, but it is challenging and requires in-depth training and practice [25,26,27]. Further, it can potentially be used by all physicians, making this technique very attractive [28]. According to a recent study, radiology residency programmes in the United States include less education periods dedicated to musculoskeletal ultrasound than residents of other specialties (i.e., physical medicine and rehabilitation, sports medicine, and rheumatology), although the established role of sonographers in the United States should be underlined [29]. On the other hand, recent studies have shown how ultrasound-guided musculoskeletal interventions are widely performed by radiologists in European countries, where ultrasound is often preferred to other imaging modalities for guiding articular injections [30,31,32,33]. Notably, the Italian College of Musculoskeletal Radiology is constantly active in supporting musculoskeletal training of radiologists involved in dedicated courses, conferences, and recommendations [34], as is also the case for the ESSR in an international setting [35,36,37]. Nevertheless, there is an urge to further improve the musculoskeletal training of young trainees to meet the demands and expectations of the radiologists of tomorrow. This issue has also been reported by a recent international survey about musculoskeletal education that highlighted how musculoskeletal training was variable in content and structure across the different countries, with limited space granted to musculoskeletal ultrasound and interventions [20,38]. In this regard, the ESSR provides a specialised qualification (European Diploma in Musculoskeletal Radiology—EDiMSK) of skills in reporting musculoskeletal examinations and interventional procedures to stimulate training of subspecialised musculoskeletal radiologists [39]. Further, the ESSR and the European Society of Radiology provide grants to young trainees to train and improve their skills through scholarships in referral centres [40]. These are just examples and possible options to enhance the musculoskeletal training of Italian board-certified radiologists and residents, supporting education through established certifications after highly specialised masters and improving national exchange programmes, with standardised fellowship programmes [41,42].

The sub-analysis of replies received from the top five most represented regions and the three macroregions was conducted due to well-known differences throughout the country, including social, economic, technological, and political differences that can be observed moving from northern to southern regions, which have an impact on universities and healthcare institutions. The sub-analysis did not show substantial differences throughout the country. Just a few differences deserve to be highlighted concerning the dedicated rotations for musculoskeletal training during residency. Indeed, it seems that a dedicated period is mostly included in Northern residency programmes (42%), particularly in Lombardia (80% of participants), where the overall rate of musculoskeletal knowledge has been reported to be higher than in the other most represented regions, where a dedicated musculoskeletal period was reported by 5–35% of participants. Notably, almost all participants from Veneto reported having no dedicated training period focused on musculoskeletal ultrasound and interventional procedures during their residency. However, regardless of the subtle discrepancies across the different schools, the vast majority of responders agreed on the need for improvement of musculoskeletal education in residency programmes.

A few limitations of this survey must be pointed out. First, this was not an all-inclusive survey, since we did not consider a number of factors related to different regional health service systems. Further, some Italian regions were under-represented, with no answers received from three regions (Valle d’Aosta, Trentino-Alto Adige, Basilicata). Nevertheless, the overall high number and response rate allowed us to provide an interesting snapshot of residents and young board-certified radiologists involved in musculoskeletal training in Italy.

## 5. Conclusions

Despite some differences in the structure of musculoskeletal education provided by different regions, there is a shared demand for improvement in musculoskeletal training, especially regarding ultrasound, MRI reporting, and interventional procedures, as proven by the large proportion of participants who perceived their musculoskeletal education as inadequate or average, highlighting the need for improvement. Recommendations and possible steps of actions should be discussed and adopted by the SIRM, and measures should be included in residency programmes. Indeed, more space should be given to musculoskeletal education in training programmes of residents, increasing the time considered for dedicated musculoskeletal rotations and the allocation of hours to musculoskeletal lessons. In particular, more time could be dedicated to some practical activities like ultrasound and interventional procedures that require active involvement from trainees. It is reasonable that not all schools of radiology may provide high-quality education on all subspecialties of radiology, including these highly specialised activities. Thus, SIRM, through its musculoskeletal section (the Italian College of Musculoskeletal Radiology), might be supportive by promoting travelling courses and educational activities to spread knowledge and skills throughout the country.

## Figures and Tables

**Table 1:** Full list of questions and answers (total participants = 1144).

Question	Total Answers	Answer
How old are you? (years)	1144/1144 (100%)	Mean: 31.4 ± 4
		(Range: 24–34)
2. Where are you attending (or have you attended) your radiology training? (Italian region)	1140/1144 (99.7%)	See Table 2
3. Are you a resident or a board-certified radiologist?	1144/1144 (100%)	Resident: 740/1144 (64.7%)
		Board-certified radiologist: 404/1144 (35.3%)
4. What type of hospital do you work in?	1142/1144 (99.8%)	University hospital: 760/1142 (66.6%)
		Public hospital: 304/1142 (26.6%)
		Private clinic: 78/1142 (6.8%)
5. During your residency, how is the MSK training organized?	1142/1144 (99.8%)	No dedicated MSK training period is scheduled in our programme: 420/1142 (36.8%)
		We learn MSK radiology scattered during our residency: 418/1142 (36.6%)
		We have a dedicated rotation for different subspecialties, included MSK radiology: 304/1142 (26.6%)
6. If a dedicated rotation in MSK radiology is scheduled, how long does it last?	300/304 (98.7%)	Between 3 and 6 months: 160/300 (53.3%)
		Less than 3 months: 96/300 (32.0%)
		Between 6 month and year: 28/300 (9.3%)
		More than 1 year: 16/300 (5.3%)
7. How is it structured?	666/1144 (58.2%)	Residents rotate among the different imaging modalities: 424/666 (63.7%)
		Residents are assigned to a specific imaging modality (e.g., MRI, ultrasound): 242/666 (36.3%)
8. Do you have a dedicated training period in MSK ultrasound during your residency?	1126/1144 (98.4%)	No: 842/1126 (74.8%)
		Yes: 284/1126 (25.2%)
9. How many hours of MSK radiology teaching are scheduled during your residency?	1070/1144 (93.5%)	<20 h: 816/1070 (76.3%)
		>40 h: 128/1070 (11.9%)
		20–40 h: 126/1070 (11.8%)
10. Who plays/played a crucial role in your MSK training?	1020/1144 (89.2%)	Senior consultants: 456/1020 (44.7%)
		Other residents: 208/1020 (20.4%)
		Junior specialists: 204/1020 (20.0%)
		University Professor: 152/1020 (14.9%)
11. How would you rate your overall MSK knowledge?	1140/1144 (99.7%)	Poor: 658/1140 (57.7%)
		Average: 250/1140 (21.9%)
		Good: 144/1140 (12.6%)
		Very good: 54/1140 (4.7%)
		Excellent: 34/1140 (3.0%)
12. How would you prefer to learn MSK radiology? (Multiple answers)	1140/1144 (99.7%)	Daily clinical practice (under supervision): 1052/1140 (92.3%)
		Dedicated courses and congresses: 544/1140 (47.7%)
		E-learning platforms: 446/1140 (39.1%)
		Clinical case-based presentations: 386/1140 (33.9%)
13. Do you have a dedicated training period in MSK interventional radiology during your residency?	1120/1144 (97.9%)	No: 950/1120 (84.8%)
		Yes: 170/1120 (15.2%)
14. How did/do you take part in interventional MSK procedures during your residency?	1000/1144 (87.4%)	No interventional MSK procedures are/were performed in my institution: 588/1000 (58.8%)
		As observer: 284/1000 (28.4%)
		Actively involved: 128/1000 (12.8%)
15. Do you think that MSK training should be improved in your residency programme?	1132/1144 (98.9%)	Yes: 1062/1132 (93.8%)
		No: 70/1132 (6.2%)
16. If yes, which area of MSK training should be improved? (Multiple answers)	1082/1144 (94.6%)	Ultrasound practice: 1004/1082 (92.8%)
		MRI interpretation and reporting during daily clinical practice: 854/1082 (78.9%)
		Ultrasound-guided procedures practice: 696/1082 (64.3%)
		Formal lectures: 654/1082 (60.4%)
		MSK case presentation and discussion: 580/1082 (53.6%)
		CT interpretation and reporting during daily clinical practice: 572/1082 (52.9%)
		CT-guided procedures practice: 506/1082 (46.8%)
		Dedicated courses and congresses: 496/1082 (45.8%)
		E-learning platforms: 450/1082 (41.6%)
		Practice in fluoroscopy-guided procedures: 350/1082 (32.4%)
17. Have you carried out/Do you think you will need to carry out a training period in a MSK dedicated center (national or foreign)?	1138/1144 (99.5%)	Yes: 776/1138 (78.2%)
		I am not sure: 214/1138 (18.8%)
		No: 148/1138 (13.0%)

**Table 2:** Number of answers stratified by the region of origin of the residency programme.

Region of the Residency Programme	Total Answers
Lombardia *	162
Sicilia ^‡^	160
Lazio ^§^	112
Toscana ^§^	112
Veneto *	108
Piemonte *	84
Emilia Romagna *	72
Campania ^‡^	58
Abruzzo ^‡^	56
Molise ^‡^	50
Umbria ^§^	46
Puglia ^‡^	42
Friuli Venezia Giulia *	30
Liguria *	16
Sardegna ^‡^	14
Calabria ^‡^	10
Marche ^§^	8

* Northern Italy; ^‡^ Southern Italy and islands; ^§^ Central Italy.

**Table 3:** The 8 most significant questions, according to the 5 most represented regions of the survey.

Question	Answers	Lombardia	Sicilia	Lazio	Toscana	Veneto
5. During your residency, how is the MSK training organized?	We have a dedicated rotation for different subspecialties, included MSK radiology	130/162 (80.3%)	18/158 (11.4%)	20/112 (17.9%)	40/112 (35.7%)	6/108 (5.5%)
	We learn MSK radiology scattered during our residency	28/162 (17.3%)	32/158 (20.3%)	50/112 (44.6%)	38/112 (33.9%)	52/108 (48.2%)
	No dedicated MSK training period is scheduled in our programme	4/162 (2.5%)	108/158 (68.3%)	42/112 (37.5%)	34/112 (30.4)	50/108 (46.3%)
6. If a dedicated rotation in MSK radiology is scheduled, how long does it last?	Between 3 and 6 months	48/130 (36.9%)	12/18 (66.7%)	14/20 (70.0%)	8/40 (20.0%)	2/6 (33.3%)
	Less than 3 months	88/130 (67.7%)	4/18 (22.2%)	6/20 (30.0%)	32/40 (80.0%)	2/6 (33.3%)
	Between 6 month and year	12/130 (9.2%)	2/18 (11.1%)	0/20 (0.0%)	0/40 (0.0%)	0/6 (0.0%)
	More than 1 year	2/130 (1.5%)	0/18 (0.0%)	0/20 (0.0%)	0/40 (0.0%)	2/6 (33.3%)
8. Do you have a dedicated training period in MSK ultrasound during your residency?	No	80/160 (50%)	120/156 (76.9%)	96/110 (87.3%)	90/110 (81.8%)	104/108 (96.3%)
	Yes	80/160 (50%)	36/156 (23.1%)	14/110 (12.7%)	20/110 (18.2%)	4/108 (3.7%)
9. How many hours of MSK radiology teaching are scheduled during your residency?	<20 h	102/160 (63.8%)	124/140 (88.6%)	84/104 (80.8%)	82/98 (83.7%)	96/104 (92.3%)
	20–40 h	24/160 (15.0%)	10/140 (7.1%)	10/104 (9.6%)	10/98 (10.2%)	4/104 (3.9%)
	>40 h	34/160 (21.2%)	6/140 (4.3%)	10/104 (9.6%)	6/98 (6.1%)	4/104 (3.9%)
11. How would you rate your overall MSK knowledge?	Poor	54/162 (33.3%)	106/158 (67.1%)	58/112 (51.8%)	86/112 (76.8%)	80/108 (74.1%)
	Average	42/162 (25.9%)	22/158 (13.9%)	28/112 (25.0%)	20/112 (17.9%)	20/108 (18.5%)
	Good	36/162 (22.2%)	18/158 (11.4%)	14/112 (12.5%)	4/112 (3.6%)	6/108 (5.6%)
	Very good	20/162 (12.4%)	8/158 (5.1%)	10/112 (8.9%)	2/112 (1.8%)	0/108 (0.0%)
	Excellent	10/162 (6.2%)	4/158 (2.5%)	2/112 (1.8%)	0/112 (0.0%)	2/108 (1.9%)
13. Do you have a dedicated training period in MSK interventional radiology during your residency?	No	120/160 (75.0%)	146/156 (93.6%)	106/112 (94.6%)	88/108 (81.5%)	106/106 (100%)
	Yes	40/160 (25.0%)	10/156 (6.4%)	6/112 (5.4%)	20/108 (18.5%)	0/106 (0.0%)
14. How did/do you take part in interventional MSK procedures during your residency?	No interventional MSK procedures are/were performed in my institution	54/152 (35.5%)	84/130 (64.6%)	80/94 (85.1%)	40/96 (41.7%)	84/94 (89.4%)
	As observer	68/152 (44.7%)	40/130 (30.7%)	10/94 (10.6%)	38/96 (39.6%)	10/94 (10.6%)
	Actively involved	30/152 (19.7%)	6/130 (4.6%)	4/94 (4.3%)	18/96 (18.8%)	0/94 (0.0%)
15. Do you think that MSK training should be improved in your residency programme?	Yes	128/158 (81.0%)	156/158 (98.7%)	110/112 (98.2%)	106/110 (96.4%)	108/108 (100%)
	No	30/158 (19.0%)	2/158 (1.3%)	2/112 (1.8%)	4/110 (3.6%)	0/108 (0.0%)

**Table 4:** The 8 most significant questions, according to the three Italian macroregions.

Question	Answers	Northern Italy, *n* = 472 (41.3%)	Central Italy, *n* = 278 (24.3%)	Southern Italy and Islands, *n* = 394 (34.4%)
5. During your residency, how is the MSK training organized?	We have a dedicated rotation for different subspecialties, included MSK radiology	198/472 (42.0%)	60/278 (21.6%)	46/392 (11.7%)
	We learn MSK radiology scattered during our residency	164/472 (34.7%)	102/278 (36.7%)	152/392 (38.8%)
	No dedicated MSK training period is scheduled in our programme	110/472 (23.3%)	116/278 (41.7%)	194/392 (49.5%)
6. If a dedicated rotation in MSK radiology is scheduled, how long does it last?	Between 3 and 6 months	62/196 (31.6%)	22/60 (36.7%)	12/44 (27.3%)
	Less than 3 months	112/196 (57.1%)	38/60 (63.3%)	10/44 (22.7%)
	Between 6 month and year	16/196 (8.2%)	0/0 (0.0%)	12/44 (27.3%)
	More than 1 year	6/196 (3.1%)	0/0 (0.0%)	10/44 (22.7%)
8. Do you have a dedicated training period in MSK ultrasound during your residency?	No	316/464 (68.1%)	234/272 (86.0%)	292/390 (74.9%)
	Yes	148/464 (31.9%)	38/272 (14.0%)	98/390 (25.1%)
9. How many hours of MSK radiology teaching are scheduled during your residency?	<20 h	340/452 (75.2%)	212/250 (84.8%)	264/368 (71.7%)
	20–40 h	56/452 (12.4%)	22/250 (8.8%)	48/368 (13.1%)
	>40 h	56/452 (12.4%)	16/250 (6.4%)	56/368 (15.2%)
11. How would you rate your overall MSK knowledge?	Poor	250/470 (53.2%)	186/278 (66.9%)	222/392 (56.6%)
	Average	110/470 (23.4%)	56/278 (20.1%)	84/392 (21.4%)
	Good	70/470 (14.9%)	22/278 (7.9%)	52/392 (13.3%)
	Very good	24/470 (5.1%)	12/278 (4.3%)	18/392 (4.6%)
	Excellent	16/470 (3.4%)	2/278 (0.7%)	16/392 (4.1%)
13. Do you have a dedicated training period in MSK interventional radiology during your residency?	No	386/460 (83.9%)	246/272 (90.4%)	318/388 (82.0%)
	Yes	74/460 (16.1%)	26/272 (9.6%)	70/388 (18.0%)
14. How did/do you take part in interventional MSK procedures during your residency?	No interventional MSK procedures are/were performed in my institution	222/426 (52.1%)	156/232 (67.2%)	210/342 (61.4%)
	As observer	142/426 (33.3%)	52/232 (22.4%)	90/342 (26.3%)
	Actively involved	62/426 (14.6%)	24/232 (10.3%)	42/342 (12.3%)
15. Do you believe that MSK training should be improved in your residency programme?	Yes	434/468 (92.7%)	268/274 (97.8%)	360/390 (92.3%)
	No	34/468 (7.3%)	6/274 (2.2%)	30/390 (7.7%)

## Data Availability

The data presented in this study are available upon reasonable request from the corresponding author.
